# A Misdiagnosed Case of Schizoaffective Disorder With Bipolar Manifestations

**DOI:** 10.7759/cureus.16686

**Published:** 2021-07-28

**Authors:** Tanya Paul, Sana Javed, Alvina Karam, Hanyou Loh, Gerardo F Ferrer

**Affiliations:** 1 Research and Academic Affairs, Larkin Health System, South Miami, USA; 2 Medicine, Avalon University School of Medicine, Willemstad, CUW; 3 Psychiatry, Nishtar Medical University, Multan, PAK; 4 Internal Medicine, Khyber Medical College, Peshawar, PAK; 5 Medicine, National University of Singapore, Singapore, SGP; 6 Psychiatry, Larkin Community Hospital, Miami, USA

**Keywords:** bipolar, psychosis, schizophrenia, schizoaffective, mania

## Abstract

Bipolar and schizoaffective disorders are both psychiatric illnesses that share common traits, but also significant differences. Due to the close overlap in symptoms, obtaining the correct diagnosis can be difficult. The management of these patients often poses a challenge to clinicians.

Five years ago, our patient was misdiagnosed with bipolar disorder with psychotic features. It was later discovered that she was suffering from schizoaffective disorder, bipolar type. The schizoaffective disorder involves symptoms of both schizophrenia and mood disorder.

## Introduction

Bipolar and schizoaffective disorders share common characteristics and because of the overlap in symptoms, getting an accurate diagnosis can be very arduous for clinicians. A comprehensive plan of previous treatment results, psychiatric and family history can help in the differentiation between these disorders [1].

Bipolar disorder is a debilitating condition and its global prevalence ranges from 1.4% to 6.4%, and individuals may experience episodes of mania, depression, and sometimes, psychotic symptoms [2]. The schizoaffective disorder occurs at a third of the rate schizophrenia does with a lifetime prevalence of 0.3% [3]. Individuals experience symptoms of psychosis, such as delusions or hallucinations and it prevails as a relatively rare disorder so the prevalence of misdiagnosis globally is not yet established. Nevertheless, psychiatrists and researchers believe there are more people who have schizoaffective disorder, the bipolar type who have been misdiagnosed with bipolar disorder with psychotic features or schizophrenia because of the similar presentation.

A cross-sectional study was held with a majority of patients suffering from psychiatric problems such as bipolar, schizophrenia, schizoaffective, and other depressive disorders. This study concluded that 39.16% of patients with psychiatric disorders were misdiagnosed, with schizoaffective disorder having the greatest rate of incidence of misdiagnosis at 75%, and bipolar disorder at 17.78% [4].

Our patient was misdiagnosed with bipolar disorder with psychotic features. On admission, with proper history taking and examinations, she was diagnosed with schizoaffective disorder, bipolar type. There are major parallels between these two disorders, but we must learn to identify key differences to provide appropriate care and treatment. Attempts have been made to better understand the incidence of misdiagnosis.

## Case presentation

A 50-year-old hypertensive, hyperglycemic, African American female presented with a history of bizarre delusions, hallucinations, disorganized behavior, and disorganized speech for the past one and a half months. The patient claimed she found three homeless in one of her beach homes, who attacked her, placing her in the hospital. She was unable to explain why she was in the psychiatric unit. On further investigation, the patient stated that she was 10 years old, a celebrity and that her father was the president of the United States. She denied any depression, homicidal, or suicidal ideations. Her past medical history was significant for diabetes type 2, hypertension, epilepsy, HIV, and acute myeloid leukemia, which are now in remission. She was previously diagnosed with bipolar type 1 disorder with psychotic features.

On physical examination, her vitals were stable and there was a scar on her right knee. When asked, the patient said the scar on her knee is a fresh wound that she got when she was attacked, before she was admitted to our hospital. When examined, the scar had properly healed, but the patient still thought she was actively bleeding. On mental status examination, her appearance was unkempt and she was poorly groomed. Her behavior was childish and very cooperative. She could name, read, repeat and comprehend, but was unable to write a complete sentence. The patient was oriented to time and place, but not to person or situation. She was euphoric and her affect was appropriate to the content. Her thought content included grandiose delusions and an overabundance of thought. Her thought process consisted of loosening of associations, flight of ideas, and tangentiality.

At the time of initial presentation, the patient was manic, easily distracted, and demonstrated aggression for not believing her. She also disrobed in front of staff and patients (indiscretion), had grandiose delusions that she was a 10-year-old child celebrity, believed she was the daughter of the American president, exhibited flight of ideas, psychomotor agitation, and pressured speech. These symptoms were also observed by the nursing home she resided in prior to her admission to the psychiatry unit. She was subsequently diagnosed with schizoaffective disorder, bipolar type according to the Diagnostic and Statistical Manual of Mental Disorders, Fifth Edition (DSM-V) criteria. The patient had delusions, hallucinations, disorganized speech, and she also exhibited grossly disorganized behavior for more than a month. These hallucinations (burglars attacking her in her beach home and seeing blood on her knee), delusions, disorganized speech, and grossly disorganized behavior were present for two weeks in the absence of her mood symptoms. Her mood episodes were reported to be present for the majority of her illness, which is why her disorder was confused with bipolar disorder with psychotic features instead of schizoaffective disorder, bipolar type. Our patient’s psychopharmacological treatment included Sertraline 50 mg once daily, Quetiapine 100 mg PO QHS, and Valproic acid 500 mg PO BID.

The patient was treated with the combination therapy of an antipsychotic with a mood stabilizer and an antidepressant. This combination therapy is used to treat 18% of schizoaffective patients. Paliperidone is the antipsychotic of choice approved by the FDA for schizoaffective disorder, but due to financial limitations, paliperidone could not be used in the treatment of our patient [3]. Quetiapine can also be prescribed in combination therapy along with a mood agent and an antidepressant for schizoaffective disorder. A social worker was consulted, and the patient was discharged to her nursing home after a week with reconciled medications, and a follow-up appointment for cognitive behavioral therapy.

## Discussion

The diagnostic criteria of schizoaffective disorder overlap greatly with many other severe psychiatric disorders seen in practice, which has made schizoaffective disorder one of the most frequently misdiagnosed psychiatric disorders [5]. Characterizing patients with psychotic and mood disorders have always proved challenging for physicians [6]. Patients like the one presented in our case report are expected to exhibit periods of mania as well as symptoms of delusions and paranoia associated with psychosis.

The continuous synchronization of psychosis and mood episodes makes it hard to distinguish mood disorders with psychotic features from schizoaffective disorder and bipolar type [6,7]. There still can be several reasons for misdiagnosis such as overlapping symptoms between bipolar disorder with psychotic features and schizoaffective disorder, bipolar type. In bipolar disorder with psychotic features, patients may experience periods of mania and symptoms of delusions [3]. Mania is defined as having at least three of the following (four if the mood is only irritable): distractibility, indiscretion, grandiosity, flight of ideas, increased activity, reduced need for sleep, and pressured speech. To be diagnosed with schizoaffective disorder, bipolar type, a person must have at least two of the following symptoms for a month, and one symptom must be from the first three listed: delusions, hallucinations, disorganized speech, grossly disorganized or catatonic behavior, and negative symptoms [3]. The second criterion required for diagnosis is that these hallucinations and delusions must be present for two weeks or more in the absence of a manic or major depressive episode [3]. Lastly, the person’s manic or major depressive episodes must be present for the majority of his or her disorder [3]. Conversely, in bipolar disorder with psychotic features, psychotic symptoms are only present during the manic phase and are the main differentiating factor between these two closely linked psychiatric illnesses [3]. This overlap of symptoms between these two psychiatric disorders was one of the core reasons why our patient remained misdiagnosed for five years.

In our case, the patient was misdiagnosed with bipolar disorder with psychotic features. After a thorough history and examination, she was diagnosed clinically with schizoaffective disorder, bipolar type. Hallucinations and delusions are prominent symptoms of schizoaffective disorder. Another symptom of schizoaffective disorder is disorganized thinking and disorganized behavior. Furthermore, for schizoaffective disorder, bipolar type, there may be periods of both depression and mania. All these symptoms were present in our patient.

Attempts have been made to better understand the incidence of misdiagnosis. The more contemporary survey conducted by National Depressive and Manic-Depressive Association (DMDA) inferred that 69% of patients with bipolar disorder were initially misdiagnosed, and more than 33% of patients remained misdiagnosed for another decade before attaining an accurate diagnosis [2]. Friedrike et al. discussed in a study that 45% of psychiatrists could not diagnose bipolar disorders because of the presence of hallucinations [8]. Another comparative study by Yuhui et al., implied schizoaffective disorder as a separate disorder, although its subtype with depressive episodes shared more similarity with schizophrenia [9].

An accurate history and physical examination are paramount to correct diagnosis, during which schizophrenia, a major depressive disorder with psychotic features, and bipolar disorder must be ruled out during the workup [10]. An observational study was conducted by Martin-Subero et al. to assess the quality of life in patients with bipolar type 1 and schizoaffective disorders. He inferred that the quality of life was poor and badly affected in all stages of both disorders. The depressed state was found to have the most impaired quality of life followed by the mixed state and manic state [11].

Schizoaffective disorder can be arduous to manage due to the manifestation of both psychotic and mood symptoms. A study by Cascade et al. had reported that 87% of patients from the SDI/Verispan’s Prescription Drug & Diagnosis Audit (PDDA) database with schizoaffective disorder, had received medications from two or more pharmaceutical classes between January 2008 and December 2008 [12]. Also, 93% received an antipsychotic and 48% received treatment for a mood disorder [12]. The most commonly prescribed treatment was an antipsychotic (22%), followed closely by an antipsychotic paired with a mood agent (20%), then an antipsychotic with an antidepressant (19%), and lastly, an antipsychotic with a mood agent and an antidepressant (18%). The rest of the possible treatment combinations have an incidence of 3% or lower and are not widely used [12].

It was reported in another article by Schnitzer et al. that combined treatment with an antipsychotic and antidepressant is an acceptable form of initial treatment for patients who have been diagnosed with schizoaffective disorder, depressive type [13]. For patients with schizoaffective disorder, bipolar type, mood stabilizers may be used instead. This is especially if the patient exhibits signs of irritability or aggression. Certain mood stabilizers, such as lithium, are thought to also be efficacious in treating the depressive subtype [13].

Several factors including an early administration and extended duration of antipsychotic medications such as paliperidone and aripiprazole have also been found to enhance the overall functioning of patients with schizoaffective disorder and schizophrenia [14]. Clozapine, on the other hand, has been found to decrease the risk of readmission to the hospital [15].

## Conclusions

Schizoaffective disorder, bipolar type, is frequently misdiagnosed in clinical practice. Psychiatrists believe that it can be avoided by getting a detailed history and proper examination in all patients with psychotic symptoms to differentiate between schizophrenia, bipolar disorder, and schizoaffective disorder. Our patient remained misdiagnosed for five years with bipolar disorder with psychotic features before reaching the correct diagnosis, due to symptoms that overlapped with other severe psychiatric disorders. In our case report, we emphasize the importance of proper examination and history taking in patients presenting with readmission and overlapping symptoms. 
